# Anti-Inflammatory Effects of *Aster incisus* through the Inhibition of NF-*κ*B, MAPK, and Akt Pathways in LPS-Stimulated RAW 264.7 Macrophages

**DOI:** 10.1155/2018/4675204

**Published:** 2018-11-29

**Authors:** Daniel Ngabire, Yeong-Ae Seong, Maheshkumar Prakash Patil, Irvine Niyonizigiye, Yong Bae Seo, Gun-Do Kim

**Affiliations:** ^1^Department of Microbiology, College of Natural Sciences, Pukyong National University, Busan, Republic of Korea; ^2^Institute of Marine Biotechnology, College of Natural Sciences, Pukyong National University, Busan, Republic of Korea

## Abstract

*Aster incisus* is a common flower found in almost all regions of South Korea. In the current study, we investigated the potential antioxidant and anti-inflammatory properties of the *Aster incisus* methanol extract in LPS-stimulated RAW 264.7 cells. We analyzed the phytochemicals contained in the extract by GC-MS. GC-MS results showed that the *Aster incisus* extract contains 9 known compounds. Later on, DPPH assay, WST-1 assay, nitric oxide (NO) assay, Western blot, and RT-PCR were conducted to investigate the anti-inflammatory effects of the extract. Our WST-1 assay results revealed that *Aster incisus* did not affect the viability of all tested cell lines up to a concentration of 200 *μ*g/ml; therefore, lower concentrations (50 *μ*g/ml and 150 *μ*g/ml) were used for further assays. *Aster incisus* scavenged DPPH and inhibited the production of NO. *Aster incisus* also reduced significantly the production of inflammation-related enzymes (iNOS, Cox-2) and cytokines (TNF*α*, IL-1*β*, and IL-6) and the gene expression of the proinflammatory cytokines. Additionally, further Western blot results indicated that *Aster incisus* inhibited the expression of p-PI3K, p-I*κ*B*α*, p-p65 NF-*κ*B, p-ERK1/2, p-SAPK/JNK, and p-Akt. Our results demonstrated that *Aster incisus* suppressed the expression of the inflammation mediators through the regulation of NF-*κ*B, MAPK, and Akt pathways.

## 1. Introduction

Inflammation is usually described as a defense mechanism used by animals to fight intruders. Inflammation can be divided into acute inflammation, which is a quick and short immune response, and chronic inflammation, which takes time to set up and may result in failure to remove the initial cause [1]. It can be related to a large number of serious diseases, like diabetes, cardiovascular disorders, Alzheimer's disease, autoimmune and pulmonary diseases, arthritis, and cancer [2].

Immune cells, especially macrophages, play a major part in the nonspecific immunity against foreign intruders, specifically infectious microorganisms (bacteria, viruses, mycoplasma, etc.) [3–5]. Their activation during chronic inflammation is very crucial and sometimes associated with complications of chronic inflammation-related diseases by excessive production of nitric oxide (NO) and prostaglandin E2 (PGE2) or other inflammation-related cytokines [6–10].

Previously published papers have described the importance of NF-*κ*B, MAPK, and Akt pathways in the expression of mediators specific to inflammation [11–14].

*Aster incisus* is a species of the Asteraceae family and belongs to the *Aster* genus which contains approximately 248 to 706 species. *Aster incisus* is native to China, Japan, and Korea [15].

Recently, we reported the anticancer effects of *Aster incisus* on gastric adenocarcinoma. [16]. Other plants belonging to the Asteraceae family have been used for traditional medicinal purposes, and most recently, plants from the Aster genus were studied and reported for their anti-inflammatory effects [17] and other biological activities [18–22].

In the current study, the potential antioxidant and anti-inflammatory effects of the *Aster incisus* extract were investigated for the first time ever in RAW 264.7 macrophages stimulated with LPS. Our results demonstrated that *Aster incisus* can inhibit LPS-induced NO and inflammation-related cytokines like TNF*α*, IL-6, and IL-1*β*. We were able to demonstrate that NF-*κ*B, MAPK, and Akt pathways were significantly inhibited by the treatment with *Aster incisus*.

## 2. Materials and Methods

### 2.1. Cell Lines and Reagents

Human kidney cells (HEK293 cells), human keratinocytes (HaCaT cells), and murine macrophages (RAW 264.7 cells) were from the ATCC (Manassas, VA, USA). DMEM media were obtained from Hyclone Laboratories (USA), and fetal bovine serum (FBS) and penicillin-streptomycin were from Cellgro (Manassas, VA, USA). The *Aster incisus* methanol extract (voucher no. 016-001) was purchased from the Korean Plant Extract Bank (KPEB, Cheongju, Korea). EZ-Cytox (WST-1; Daeil Lab Service, Seoul, Korea), dimethyl sulfoxide (DMSO; Sigma, St. Louis, MO, USA), LPS (Sigma, MO), Griess reagent (Sigma, USA), and cell lysis buffer were purchased from Intron Biotechnology Inc., Gyeonggi, Korea. The first antibodies (iNOS, Cox-2, TNF*α*, pI*κ*B*α*, I*κ*B*α*, p-p65 NF*κ*B, NF*κ*B, p-Akt, p-PI3K, p-mTOR, p-SAPK/JNK, p-ERK1/2, and p-p38) and the second antibody linked to a peroxidase were purchased from Cell Signaling Technology (CST; Danvers, MA, USA). ECL detection solution was obtained from Pierce (Rockford, IL, USA) and 4% formaldehyde from Sigma (Sigma-Aldrich, St. Louis, MO, USA) while rabbit normal serum and anti-rabbit IgG were purchased from Cell Signaling Technology (CST; Danvers, MA, USA).

### 2.2. Gas Chromatography-Mass Spectroscopy (GS-MS)

The *Aster incisus* methanol extract (AIE) sample obtained from the Korean Plant Extract Bank (voucher no. 016-001) was analyzed using GC-MS equipment. The experimental settings were as follows: the standard nonpolar column dimensions were 30 × 0.25 *μ*m ID × 0.25 *μ*m df and helium was used as a gas carrier at a flow rate of 1.0 ml/min. The oven temperature was set at 50°C and later raised to 320°C at a speed of 7°C/min. The temperature for the injector was fixed at 280°C with the volume of the injection equal to 0.1 *μ*l. The interpretation was conducted using the database of the NIST library.

### 2.3. DPPH Assay

The antioxidant scavenging activity was investigated by using the scavenger and free radical 2,2-diphenyl-1-picrylhydrazyl (DPPH). This experiment was conducted as detailed by Patil et al. [23] with minor variations. In brief, 1 ml from various concentrations of AIE diluted in methanol (20, 50, 100, and 150 *μ*g/ml) was mixed with 4 ml of methanol each containing 0.07 mM of DPPH. Afterwards, the mixtures were vigorously vortexed followed by an incubation in the dark at room temperature for about 30 min. The DPPH scavenging result data were collected by reading the absorbance at 517 nm. For standard, we used ascorbic acid and methanol as a blank. The inhibition percentage was determined using the following formula:

(1) $$\begin{array}{c} Percentage\text{ of }inhibition=\left[\frac{abs\text{ of }control–abs\text{ of }samples}{control\;abs}\right]\times 100. \end{array}$$

All scavenging analyses were conducted in triplicate, and the values are expressed as the result mean values ± standard deviation (SD).

### 2.4. Cell Culture

HaCaT cells, HEK 293 cells, and RAW 264.7 cells were obtained from ATCC. They were cultured in DMEM medium containing 10% of FBS and 1% of antibiotics (penicillin-streptomycin) and subcultured every time they reached 80–90% of confluency for HaCaT cells and HEK 293 cells and 70% of confluency for RAW 264.7 cells. All cells were incubated at 37°C and 5% of CO_2_.

### 2.5. Cell Cytotoxicity

The toxicity of AIE was analyzed using the WST-1 reagent after the treatment of HaCaT cells, HEK 293 cells, and RAW 264.7 cells with various concentrations of AIE. All cell lines were plated in 96-well plates separately at a concentration of 1 × 10^4^ cells per well in 100 *μ*l of DMEM overnight. After 24 h of incubation, cells were treated with different concentrations of AIE (100, 150, and 200 *μ*g/ml) for 24 h. After treatment, the supernatant in each well was removed and replaced by fresh prewarmed media. Additionally, 10 *μ*l of the WST-1 reagent was added in each well and the plates were incubated in the dark at 37°C for further 3 h. After incubation, viable cells were quantified using an ELISA microplate reader by measuring the absorbance at 460 nm.

### 2.6. NO Assay

For the NO production, RAW 264.7 macrophages were cultured in a 24-well plate for 24 h at a final concentration of 5 × 10^4^ cells per well overnight. After 24 h of incubation, RAW 264.7 cells were later challenged with 50 *μ*g/ml and 150 *μ*g/ml of AIE for 4 h and then stimulated with 1 *μ*g/ml LPS for further 24 h. The produced NO was established by quantifying the nitrate accumulated in the collected supernatant in which we added a 1 : 1 volume of Griess reagent. The ELISA microplate reader was used for the quantification of NO at 540 nm.

### 2.7. Western Blot

Initially, RAW 264.7 cells were plated in 100 mm dishes and incubated for 24 h or 36 h at 37°C. The next day, they were treated with AIE (50 and 150 *μ*g/ml) for 4 h prior to a 30 min or 18 h stimulation period with LPS (1 *μ*g/ml). For the protein extraction, cells were collected in PBS and lysed in an ice-cold lysis buffer. The concentration of proteins in each sample was measured using the Bradford protein assay. During separation in gel electrophoresis, 30 *μ*g of protein mixture from each sample was loaded in the wells of 12% polyacrylamide gels. Once the electrophoresis was finished, the proteins in the gels were transferred onto Western blot nitrocellulose membranes for 2 h at 50 volts. After the transfer, both sides of the membranes were blocked with 5% of skim milk diluted in PBS-Tween 20 (PBST) for 1 h. The membranes were then washed with PBST three times and incubated with diluted antibodies (p38 MAPK, GADPH, p-SAPK/JNK, Cox-2, TNF*α*, p-Akt, p-PI3K p85, PI3K p85, p-p38 MAPK, p-ERK1/2, SAPK/JNK, ERK1/2, p-I*κ*B*α*, Akt, I*κ*B*α* iNOS, NF-*κ*B, and p-p65 NF-*κ*B (Ser536)) overnight. All the used antibodies were obtained from CST (Cell Signaling Technology). After three consecutive washings, the membranes were incubated with secondary antibodies conjugated with peroxidase for 1 h and a half, and the protein bands were revealed in a dark room.

### 2.8. RT-PCR

RAW 264.7 cells were cultured in DMEM and incubated at 37°C for 24 h. The following day, cells were treated with 50 and 150 *μ*g/ml of AIE for 4 h and then stimulated with LPS for 6 h. Total mRNA from RAW 264.7 cells was extracted using a Qiagen RNeasy plus kit as described by the manufacturer. Total mRNA (2 *μ*g) was converted to cDNA in an equal series of standard 10 *μ*l reverse transcription reactions. Obtained DNA was amplified by PCR reactions. Primer sequences that were used to amplify the targeted cDNA fragment are presented in Table 1. For all amplification steps, we run 30 cycles with each made by the following steps: DNA denaturing at 94°C for 30 s, primer pairing at 57°C for 30 s, and primer elongation at 72°C for 30 s. The obtained cDNA fragments were then separated in a 1.2% agarose gel for 15 min by electrophoresis at 100 V and further revealed under UV light right after the gels were stained with ethidium bromide. GAPDH bands were used as a reference for specific gene targets.

### 2.9. Immunofluorescence

RAW 264.7 cells were seeded at 2 × 10^5^ cells/well on cover glass bottom dishes for 24 h. After 24 h incubation, cells were treated or not with AIE for 4 h and then stimulated or not with LPS for 30 min. Following stimulation and treatment, the cover glass dishes were washed each twice with phosphate-buffered saline (PBS), stained with 1 *μ*M 4,6-diamidino-2-phenylindole (DAPI; Thermo Scientific, Rockford, IL) diluted in methanol for 20 min at 37°C, and washed twice with PBS after staining. After blocking for 2 hours with 5% dry skim milk, cells were incubated with an anti-p65 primary antibody at 4°C overnight. The following day, dishes were washed twice with PBS, and an Alexa Fluor 555-conjugated secondary antibody was added for 2 hours. After incubation with the secondary antibody, the cover glass bottom dishes were washed with PBS and coverslips were mounted on the slides. Cells were visualized under an LSM 510 laser confocal microscope from Zeiss (Jena, Germany).

### 2.10. Statistical Analysis

All the experiments in this study were repeated three times before the analysis. The statistical analysis of the obtained data was conducted using GraphPad Prism7 (GraphPad Software, San Diego, CA). A probability value of *P* < 0.05 was considered significant. Our analyzed data are presented as mean value ± standard deviation (SD).

## 3. Results

### 3.1. Gas Chromatography-Mass Spectrum Analysis

**Figure 1:**
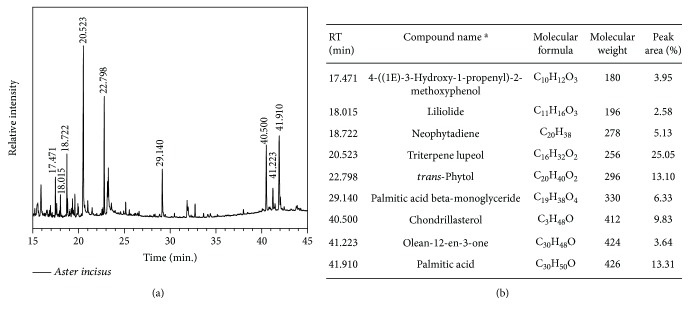
(a) Gas chromatography-mass spectrometry of the *Aster incisus* methanol extract. (b) Chemical compounds identified in the methanol extract of *Aster incisus*.

GC-MS analysis of the methanol extract of *Aster incisus* showed 9 peaks (Figure 1(a)) which indicated the presence of 9 phytochemical constituents (Figure 1(b)). After comparison with the NIST library, the 9 compounds were identified and characterized. 4-((1E)-3-Hydroxy-1-propenyl)-2-methoxyphenol (3.95%), liliolide (2.58%), neophytadiene (5.13%), triterpene lupeol (25.05%), *trans*-phytol (13.10%), palmitic acid beta-monoglyceride (6.33%), chondrillasterol (9.83%), olean-12-en-3-one (3.64%), and palmitic acid (13.31%) were present in the extract.

### 3.2. Antioxidant Capacities of AIE

**Figure 2:**
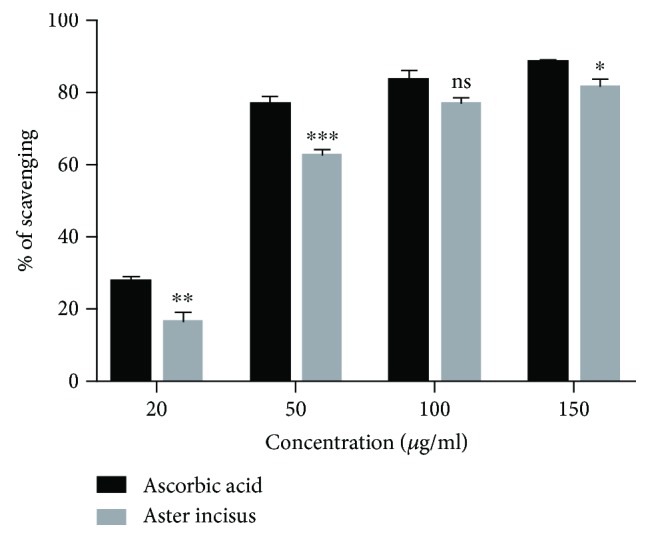
DPPH scavenging activity of *Aster incisus*. DPPH scavenging graph of *Aster incisus* and of the standard, ascorbic acid. The image displays the scavenging percentage of the DPPH radical by *Aster incisus*. Statistical differences between ascorbic acid and Aster incisus were significant at the values of ^∗^*P* < 0.05, ^∗∗^*P* < 0.01, or ^∗∗∗^*P* < 0.001.

The scavenging capacity of AIE was assimilated with ascorbic acid for comparison as the standard antioxidant. The results in Figure 2 are a representation of the radical scavenging abilities of the AIE compared to the standard. In our results, the values of radical scavenging activity for DPPH were found to be 18.28%, 61.60%, 78.17%, and 80.07% for 20, 50, 100, and 150 *μ*g/ml of AIE, respectively (Figure 2).

### 3.3. AIE Effects on Cell Viability

**Figure 3:**
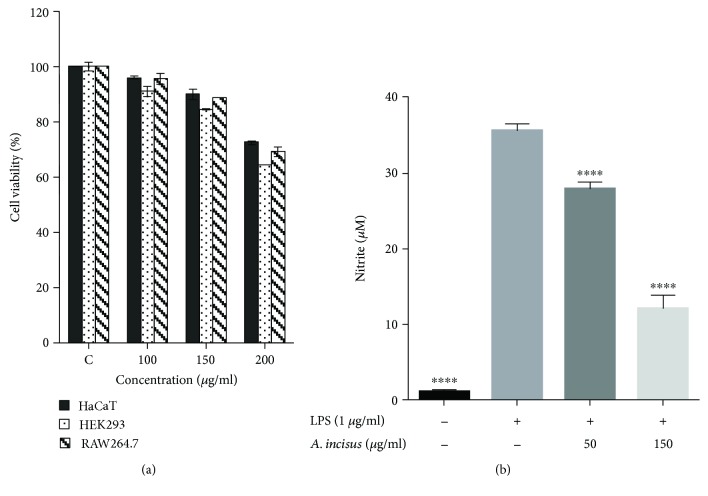
*Aster incisus* cytotoxicity and effects on nitric oxide (NO) production. (a) HaCaT, HEK293, and RAW 264.7 cells were cultured for 24 h and treated with *Aster incisus* as shown above, and cell viability was obtained by the WST-1 assay. (b) After 24 h incubation of RAW 264.7 cells, the macrophages were treated with *Aster incisus* for 4 h followed by stimulation with 1 *μ*g/ml LPS for further 24 h. NO concentrations were determined using the Griess reagent. Statistical differences between the treatment groups and the control group compared to the LPS-stimulated nontreated group were significant at a value of ^∗∗∗∗^ *P* < 0.0001.

The cytotoxicity effect of AIE was measured using the WST-1 assay in RAW 264.7 cells, HaCaT cells, and HEK 293 cells. As shown Figure 3(a), AIE did not affect the cell viability after 24 h treatment. However, AIE was cytotoxic at an increased concentration of 200 *μ*g/ml. These results were used to determine the safe concentrations for further experiments; therefore, in the subsequent experiments, AIE was used at concentrations up to 150 *μ*g/ml.

### 3.4. Inhibition Nitric Oxide Production by AIE

The effects of AIE on the production of nitric oxide in the supernatant media of RAW 264.7 cells were investigated and determined 24 hours after cells were treated with 1 *μ*g/ml LPS and different concentrations of AIE (50 and 150 *μ*g/ml). As shown in Figure 3(b), AIE significantly decreased the production of nitric oxide. We can conclude from these results that AIE can inhibit in RAW 264.7 macrophages the production of nitric oxide when stimulated with LPS.

### 3.5. Inhibition of Inflammation Mediators by AIE

**Figure 4:**
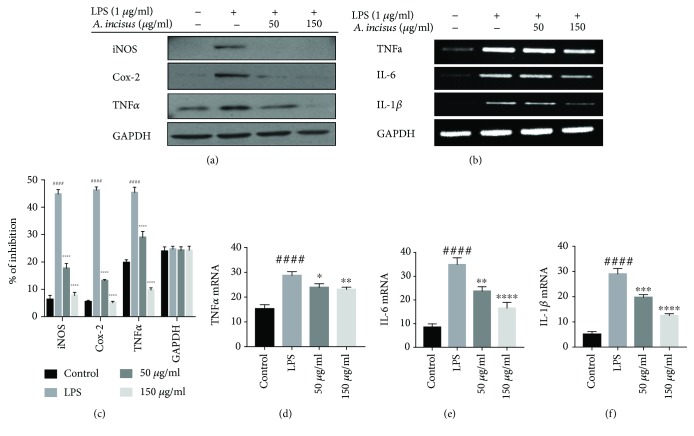
*Aster incisus* effects on inflammatory enzymes and cytokines. (a) Levels of iNOS and Cox-2 enzymes and of cytokine TNF*α* were determined using Western blot analysis after cell treatment with *Aster incisus* and activation with LPS. (b) RAW 264.7 macrophage cells were treated with *Aster incisus* for 4 h and activated with LPS for 6 h. Messenger RNA (mRNA) expression of cytokines like TNF*α*, IL-6, and IL-1*β* was analyzed by RT-PCR. (c) Western blot analysis of iNOS, Cox-2, and TNF*α.* (d) The expression of TNF*α* mRNA. (e) The expression of IL-6 mRNA. (f) The mRNA levels of IL-1*β*. Differences among different treatment groups and the LPS-stimulated nontreated group were significant at the values of ^∗^*P* < 0.05, ^∗∗^*P* < 0.01, ^∗∗∗^*P* < 0.001, or ^∗∗∗∗^ *P* < 0.0001. The statistical difference among the control and the LPS-stimulated nontreated groups was considered significant at a value of ^####^*P* < 0.0001.

In addition to NO production investigation, we further analyzed the action of AIE in inflammation-related enzymes iNOS and Cox-2 and the expression of cytokines. The Western blot analysis of iNOS, Cox-2, and TNF*α* (Figure 4(a)) showed a significant reduction in their expression. After the investigation on the expression of these inflammatory mediators, we continued by conducting RT-PCR on TNF*α*, IL-6, IL-1*β*, and GAPDH mRNAs (Figure 4(b)) showing that AIE did also significantly inhibit the expression of cytokines at the mRNA level. These results suggest that AIE can regulate the gene expression of proinflammatory cytokines.

### 3.6. Inhibition of NF-*κ*B Phosphorylation and Translocation in LPS by AIE

**Figure 5:**
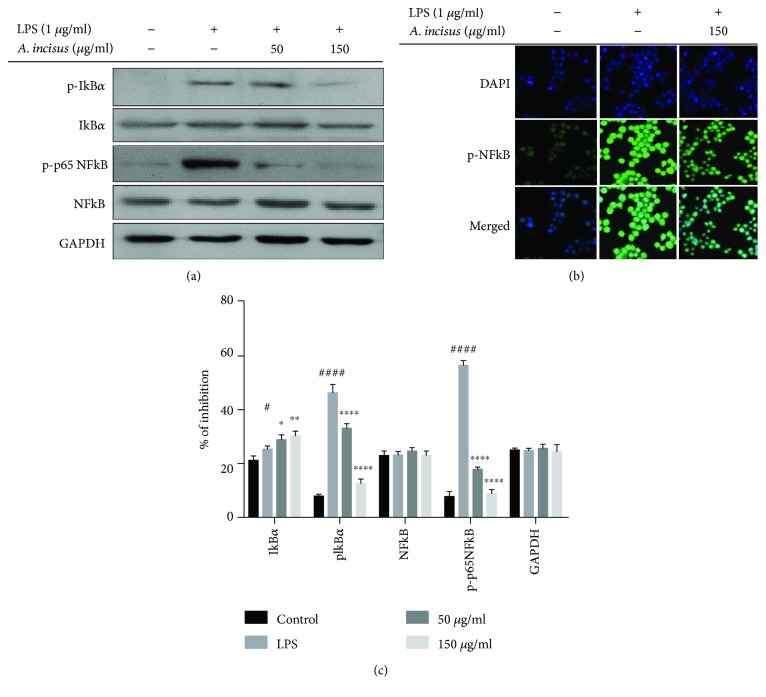
Effects of *Aster incisus* on the NF-*κ*B pathway. (a) For NF-*κ*B protein expression, RAW 264.7 macrophage cells were cultured for 36 h, treated with *Aster incisus* for 4 h, and activated with LPS for 30 min, and whole cell lysates were separated by Western blot. (b) p-p65 translocation was analyzed by immunofluorescence, and cultured RAW 264.7 macrophage cells were treated with *Aster incisus* for 4 h and activated with LPS for 30 min. DAPI nuclear staining and anti-p-p65 NF-*κ*B were used for p-p65 localization. (c) Western blot levels of I*κ*B*α*, p-I*κ*B*α*, NF-*κ*B, and p-p65 NF-*κ*B. Statistical differences between groups treated with different concentrations and the LPS-stimulated nontreated group were significant at the values of ^∗^*P* < 0.05, ^∗∗^*P* < 0.01, or ^∗∗∗∗^ *P* < 0.0001. The statistical difference among the control and the LPS-stimulated nontreated groups was significant at the values of ^#^*P* < 0.05 or ^####^*P* < 0.0001.

NF-*κ*B proteins are heterodimers represented by two monomers p50 and p65. Their activation is prevented by I*κ*B*α*. The activation of I*κ*B*α* by phosphorylation induces the translocation of phosphorylated NF-*κ*B from the cytoplasm into the nucleus for the regulation of specific transcription factors. To investigate the effect of AIE on the regulation of the NF-*κ*B pathway, the p-NF-*κ*B protein and p-I*κ*B*α* were examined using Western blotting analysis. As shown in Figure 5(a), p-NF-*κ*B and p-I*κ*B*α* were negatively regulated in a dose-dependent manner; then, further p-NF-*κ*B was visualized using immunofluorescence to evaluate the effect of AIE on its translocation. The results in Figure 5(b) showed that AIE inhibited p-NF-*κ*B translocation from the cytoplasm to the nucleus. These results reveal that AIE regulates the inflammatory response of RAW 264.7 cells by inhibiting the phosphorylation of p-NF-*κ*B and p-I*κ*B*α*.

### 3.7. AIE Inhibited MAPK Pathway Activation

**Figure 6:**
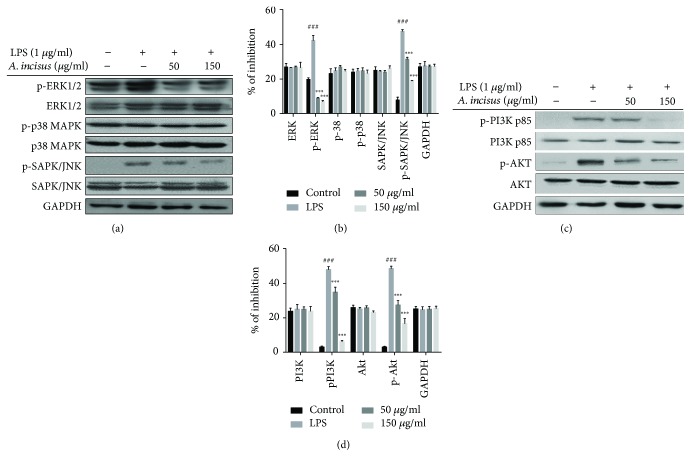
*Aster incisus* effects on the MAPK pathway. RAW 264.7 macrophages cell were cultured in DMEM, treated with *Aster incisus*, and activated with LPS, and whole cell lysates were obtained and proteins were quantified before being separated by Western blot. The phosphorylated and nonphosphorylated proteins of the MAPK kinase pathway were analyzed as shown above. (b) *Aster incisus* effects on the PI3K-Akt pathway. RAW 264.7 macrophage cells were treated with *Aster incisus* for 24 h and activated with LPS for 30 min, followed by protein electrophoresis. These cells were viewed by Western blot. The phosphorylated and nonphosphorylated proteins of the Akt pathway were analyzed using specific antibodies, and GAPDH was used as a standard protein. Differences between treatment groups and the LPS-stimulated nontreated group were considered significant at a value of ^∗∗∗^*P* < 0.001. The statistical difference among the control and the LPS-stimulated nontreated groups was significant at a value of ^###^*P* < 0.001.

As MAPK proteins are also crucial to NO and proinflammatory cytokine production and are therefore potential efficient targets of AIE, we later examined the function of MAPK pathway proteins in the inhibition of NO and cytokines by AIE. RAW 264.7 macrophage cells were initially treated with AIE for 4 h and later on stimulated with LPS for 30 min. The phosphorylated forms of ERK1/2, p38, and SAPK/JNK were strongly increased by the LPS treatment, but when cells were treated with AIE after LPS activation, ERK1/2 phosphorylation and SAPK/JNK phosphorylation were decreased in a dose-dependent manner (Figure 6(a)). Western blot of p-p38 MAPK did not show any significant inhibition of the protein expression in treated LPS-activated RAW 264.7 macrophages.

### 3.8. AIE Inhibited PI3K/Akt Pathway Activation

Activation of the PI3K/Akt pathway results in the regulation of numerous important cell processes including the production of the main inflammation-related mediators. Our study investigated the effects of AIE treatment on the expression of PI3K and Akt proteins in RAW 264.7 macrophages activated with LPS using Western blot analysis. Our results revealed that AIE suppressed the LPS-induced phosphorylation of PI3K and Akt (Figure 6(b)).

## 4. Discussions

Herbal medicines have been used for decades as remedies for a wide range of diseases. In recent years, published studies showed that plants have various biological activities including anti-inflammatory effects through the regulation or inhibition of inflammatory mediators such as cytokines and pathways involved in their production [24, 25].

Inflammation is defined as an immune defensive response to infection or injury and has been shown to play a crucial and pivotal role in a variety of diseases. When the body fails to resolve the inflammation, tissue injury and loss of function can occur. Inflammation plays a major role in the complications of many diseases like inflammatory bowel diseases (IBD), atherosclerosis, and cancer. It is mostly associated with a bad prognosis, and when not well treated, it can precipitate patient death. Inflammation is amplified by the production of inflammatory cytokines that mostly recruit and attract other cells to the site of inflammation [26]. Additionally, free radicals like NO and H_2_O_2_ are being produced during inflammation and can lead to vasodilatation and cell damage that in the end will also amplify inflammation; therefore, the inhibition or downregulation of the inflammatory mediators is a major focus in the search for new anti-inflammatory molecules. In this study, we analyzed AIE with GC-MS. The results indicate that AIE contained 9 known phytochemicals. We evaluated the antioxidant and anti-inflammatory activities of AIE in LPS-activated RAW 264.7 murine macrophages. Results from our study demonstrated that AIE significantly scavenged DPPH and inhibited the production of NO together with the downregulation of iNOS enzyme expression.

Inflammatory molecules like prostaglandins and cytokines have all been shown to be supporting the malignant phenotype in cancer development. Highly expressed IL-1*β* has been presented in many human cancers such as melanoma and lung, breast, colon, neck, and head cancers. TNF*α* is destructive to tumor blood vessels and induces cell death by necrosis at high concentrations while elevated levels of IL-6 are also present in numerous tumors such as colorectal cancer, gastric carcinoma, and Hodgkin lymphoma. Considering all the data accumulated about inflammatory cytokines and their function in many pathologies specifically in cancer, it is therefore very important to control inflammation by finding more efficient compounds that can inhibit triggered inflammation [26–29]. In the current study, we investigated the effects of AIE in RAW 264.7 cells stimulated with LPS and AIE markedly suppressed the release and expression of IL-6, IL-1*β*, and TNF*α* [27–30].

To further investigate the anti-inflammatory function of plants, we need to understand pathways related to the production of inflammatory mediators. Numerous studies demonstrated that specific transcription factors are responsible for the regulation of a large number of molecules and proteins from activated macrophages. The NF-*κ*B transcription factor family is a crucial pathway in inflammatory response processes; therefore, inhibition of activated NF-*κ*B units in the immune defense system is nowadays considered to be a major therapeutic target for the decrease in intense inflammatory responses. Besides NF-*κ*B pathway activation, MAPK and Akt pathways have also been confirmed to be major players in the expression of numerous proinflammatory genes [31, 32]. After stimulation with LPS through TLR4, proteins from these pathways are activated by phosphorylation and their activated forms can further regulate their specific transcription factor targets. Therefore, both pathway signaling cascades are therapeutic targets for the development and production of efficient anti-inflammatory substances. Our results revealed that AIE partially inhibited phosphorylation of MAPK proteins: ERK and SAPK/JNK, but not p38 MAPK. We also were able to find that AIE attenuated the expression of the phosphorylated p65 protein and the degradation of p-I*κ*B*α*. Immunofluorescence images showed that AIE is able to regulate the NF-*κ*B pathway in another way by blocking the nuclear translocation of the phosphorylated p65 [33–37].

**Figure 7:**
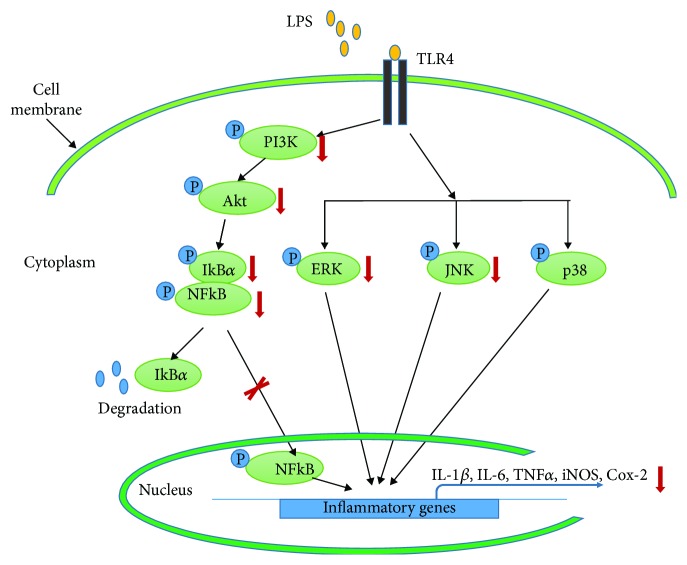
Schematic representation of the inhibition mechanisms by *Aster incisus* in LPS-induced inflammation.

In conclusion, as actual scientific studies have shown the importance of controlling inflammation in various diseases, we investigated the potential antioxidant and anti-inflammatory effects of AIE in RAW 264.7 cells stimulated with LPS. As shown in Figure 7, AIE successfully inhibited proinflammatory cytokines via 3 different pathways. Our results demonstrated that AIE is a promising source of anti-inflammatory compounds.

## Figures and Tables

**Table 1:** PCR primers of inflammatory markers.

Primer	Sequence	
	Forward	Reverse
IL-1*β*	5′-ATGGCAACTGTTCCTGAACTCAACT-3′	5′-TTTCCTTTCTTAGATATGGACAGGAC-3′
IL-6	5′-AGTTGCCTTCTTGGGACTGA-3′	5′-CAGAATTGCCATTGCACAAC-3′
TNF*α*	5′-ATGAGCACAGAAAGCATGATC-3′	5′-TACAGGCTTGTCACTCGAATT-3′
GAPDH	5′-TGAAGGTCGGTGTGAACGGATTTGGC-3′	5′-CATGTAGGCCATGAGGTCCACCAC-3′

## Data Availability

The data used to support the findings of this study are available from the corresponding author upon request.
